# New insights into the stromal interaction molecule 2 function and its impact on the immunomodulation of tumor microenvironment

**DOI:** 10.1186/s13578-024-01292-8

**Published:** 2024-09-13

**Authors:** Shishan Zhou, Shujie Liu, Anfeng Jiang, Zhiyuan Li, Chaojun Duan, Bin Li

**Affiliations:** 1 Department of Oncology, Xiangya Hospital, Central South University, Changsha, 410008 Hunan People’s Republic of China; 2 Department of Thoracic Surgery, Xiangya Hospital, Central South University, Changsha, 410008 Hunan People’s Republic of China; 3 Institute of Medical Sciences, Xiangya Hospital, Central South University, Changsha, 410008 Hunan People’s Republic of China; 4 National Clinical Research Center for Geriatric Disorders, Changsha, 410008 Hunan People’s Republic of China

**Keywords:** STIM2, Function, Immunomodulation, Tumor microenvironment

## Abstract

Immune cells-enhanced immunotherapy exhibits unprecedented overall survival-prolongation even curable in some cancer patients. Although so, most of the patients show no response. Tumor microenvironment (TME) where immune cells settle down has multi-faceted influences, but usually creates an immunosuppressive niche that facilitating tumor cells escape from immune attack. The metabolites and malnutrition of TME exert enormous effects on the resident immune cells, but the underlying mechanism is largely unknown. The stromal interaction molecules 2 (STIM2) is an endoplasmic reticulum (ER) calcium (Ca^2+)^ sensor to maintain Ca^2+^ homeostasis. Notably, the cytosol STIM2 C-terminus is long with various domains that are available for the combination or/and molecular modification. This distinct structure endows STIM2 with a high susceptibility to numerous permeable physico-chemical molecules or protein interactions. STIM2 and its variants are extensively expressed in various immune cells, especially in T immune cells. STIM2 was reported closely correlated with the function of immune cells via regulating Ca^2+^ signaling, energy metabolism and cell fitness. Herein, we sum the latest findings on the STIM2 structure, focusing on its distinct characteristics and profound effect on the regulation of Ca^2+^ homeostasis and multi-talented functionality. We also outline the advancements on the underlying mechanism how STIM2 anomalies influence the function of immune cells and on the turbulent expression or/and amenably modification of STIM2 within the tumor niches. Then we discuss the translation of these researches into antitumor approaches, emphasizing the potential of STIM2 as a therapeutic target for direct inhibition of tumor cells or more activation towards immune cells driving to flare TME. This review is an update on STIM2, aiming to rationalize the potential of STIM2 as a therapeutic target for immunomodulation, engaging immune cells to exert the utmost anti-tumor effect.

## Introduction

Nowadays, tumor remains one of the biggest threats to human health. Hanahan et al. have proposed that tumor cells are characteristic of multiple hallmarks. Some of them, such as triggering tissue inflammation and activating invasion are responsible for function-impaired organs and high fatality in patients [1]. Although highly heterogeneous, tumor cells are widely accepted as the emanation of normal cells which undertake the repurposing of physiological signalings. At the cellular level, Ca^2+^ is an explicit messenger that controls a host of downstream signaling events that are triggered by intrinsic stimuli, environmental irritation and oncogenic signaling. Available evidence indicates that the alterations in the Ca^2+^ toolkits have been engaged in the pathogenesis of various types of tumors such as colon cancer, breast cancer, glioma, melanoma and sarcoma [2–4].

Currently, one of the most successful strategies is T-cell-central immunotherapy, which has come to clinical practice in a broad spectrum of types of tumors, whereas the overall response was usually below 20%. Other immune cells including B lymphocytes, macrophages, and neutrophils also play important roles in immune attack [5]. Many studies have demonstrated that Ca^2+^ signaling is crucial for the competence of immune cells to fulfill these functions, and its dysregulation exhibits incredible impacts on the functions of immune cells thus causing immune compromise diseases such as autoimmune or immune-deficiency disease.

The endoplasmic reticulum (ER) is the biggest intercellular Ca^2+^ store. In response to intrinsic or extrinsic stimuli, ER Ca^2+^ flows into the cytosol for the allowance of innumerable cellular molecule events, and then ER Ca^2+^ should be refilled mostly through CRAC complex which mainly consists of the plasma membrane (PM)-resided ORAI calcium release-activated calcium modulator 1–3 (ORAI1-3) and the stromal interaction molecule 1–2 (STIM1-2) with N-terminal located on ER while the long C-terminal facing the plasma. Currently, two STIM isoforms, STIM1 and STIM2 were discovered in humans which were initially studied and characterized in the T lymphocytes and mast cells [6]. Recently, the novel structures or isoforms and the explicable functions of STIM2 have been unraveled on Ca^2+^ homeostasis, adaption to metabolism reprogram, as well as its cytosol accountable interface modulated by the soluble factors within the tumor microenvironment (TME) [7]. These findings provide the potential of STIM2 as a target toward immunomodulation that could be the combination strategy with current IM, on its inhibition or activation.

## Overview of STIM2 structure

**Fig. 1:**
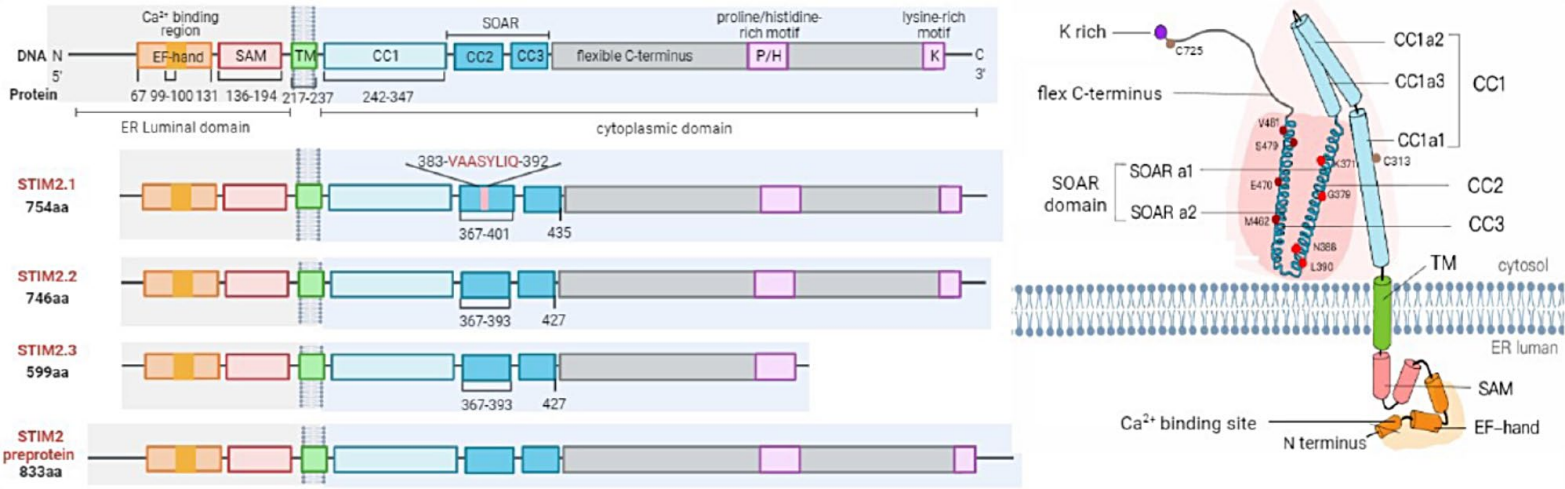
The structure of STIM2. Plain diagram (left) of STIM2: STIM2 includes ER luminal N-terminal region (EF-hand, SAM), single TM domain and cytoplasmic C-terminal region (CC1, CC2, CC3, CAD/SOAR domain, P/H motif, K motif). STIM2 has 3 splicing variants, STIM2.1 with an insert of amino acids (383-VAASYLIO-392) in CAD/SOAR domain, STIM2.2 and STIM2.3. STIM2 preprotein produced by inefficient cleavage of the signal peptide. Dimensional diagram (right) of STIM2: CA2 + binding site of N-terminus is shown in orange, SOAR domain for the interaction of STIM with ORAI is shown in pink, where the key residues of K371/G379/N388/L390 in SOARa1, and M462/E470/S479/V481 in SOARa2 as shown with red and dark red dot, respectively

In 2001, the STIM2 gene was first described as a homolog of STIM1 in mammals. It is located at chromosome 4p15.1, and has 11 introns and 12 exons, consisting of 833 amino acid residues (105–115 kDa). The overall protein structure of STIM2 is similar to STIM1, but 100 aa longer with a few differences [8]. It includes an N-terminal, transmembrane (TM) and C-terminal with a single TM structure. During the translation, Met1-Gly101 peptide in the N-terminus will be cleaved by a signaling peptide peptidase (SPP), then producing STIM2-Fc, most of which was translocated to ER for glycosylation while a small stable portion remains in the cytosol with signal peptide intact and unglycosylated, named as pre-STIM2 Fc. The met1-Gly 101 signal peptide was further cleaved by SPP and released an 88-91-aa signal peptide fraction (SPF) into the cytosol [9]. Thus, endogenous STIM2 could exist concomitantly in three distinct forms during its translation process to STIM2-Fc, including ER resident STIM2, cytosolic preSTIM2 and cytosolic SPF [10] (Fig. 1).

Generally, the ER-resident STIM2 consists of canonical EF-hand (cEF) domains, hidden or noncanonical EF-hand (hEF) domains and a sterile motif (SAM). The cEF is the effective Ca^2+^-binding domain while hEF and SAM contribute to the conformation alteration in response to the decrease of ER Ca^2+^ concentration. The following structure is TM domain, marking the end of the N-terminal portion. The C-terminal part of STIM2 includes the CC1 domain, CC2 and CC3 domains [11–13]. The CC1 domain directly connects to the TM domain, while the CC2 and CC3 domains are referred to as the CRAC-activating domain (CAD) or STIM-ORAI-activating region (SOAR) that facilitate the extension of the C-terminal domain toward the PM and direct interaction via the conserved lysine residues with PM phosphoinositides (PIPs) to trap STIM2 at ER-PM membrane contact sites.

The CC1 domain is further subdivided into three α-helices named α1, α2, and α3, while CAD/SOAR is separated into four helical regions named Sα1, Sα2, Sα3, and Sα4. The end of the C-terminus in the cytoplasm is proline-rich domains and lysine-rich domains which is a multifaceted interaction region, one of which is for anchoring the activated STIM proteins within ER-PM junctions where the SOAR domain is available for the recruitment and activation of ORAI1 [14].

## STIM2 biological function

**Fig. 2:**
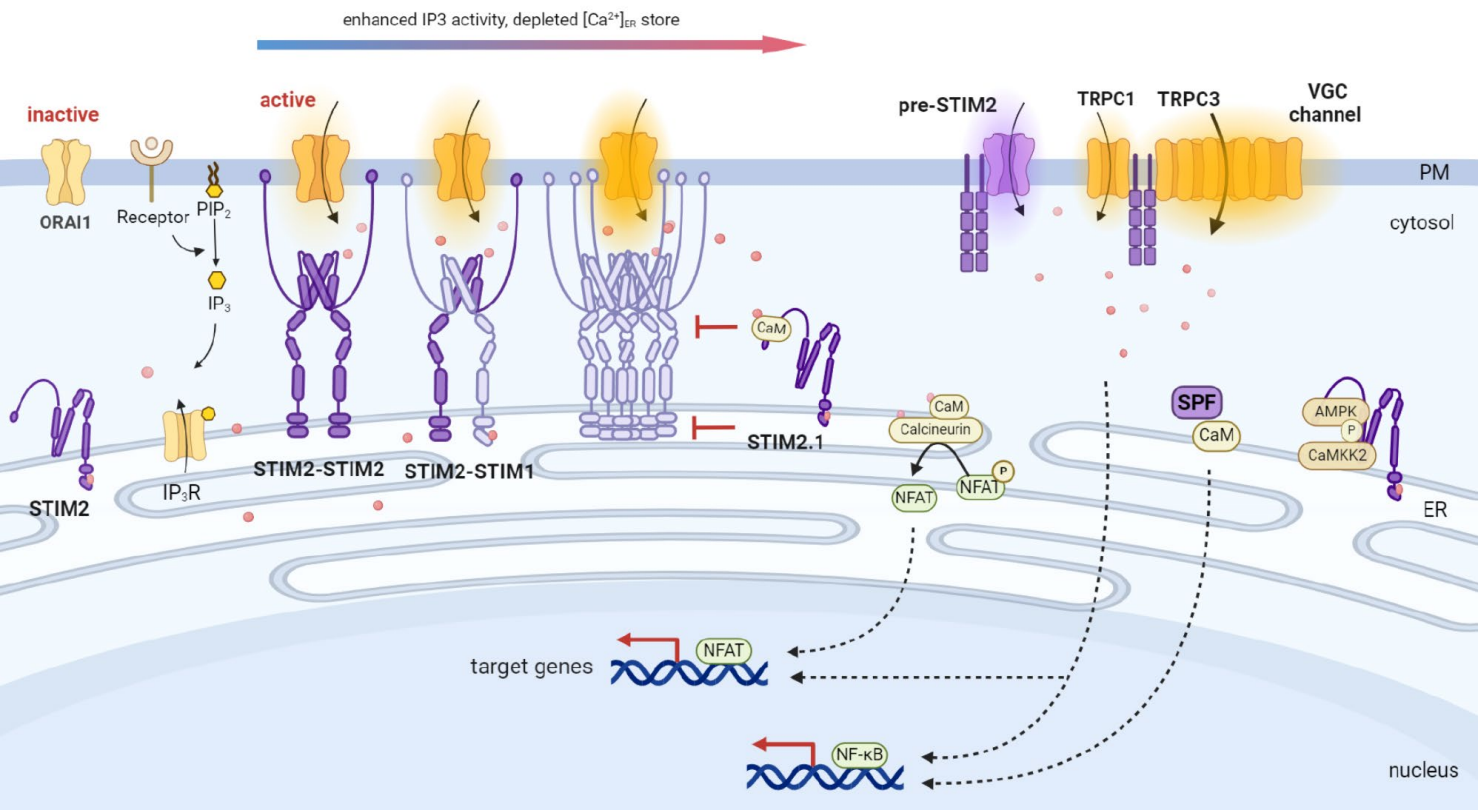
Overview of STIM2 role on Ca2 + homeostasis and other biology functions. In resting conditions, STIM2 was recruited to ER-PM junctions mostly gating ORAI1 in imobile status upon the IP3 and IP3R drived by PLC-dependent PIP2 hydrolysis and cAMP/PKA activity. Upon minimal depletion of [Ca^2+^]_ER_, STIM2 oligomerizes and binds to ORAI1 in ER-PM junction to mediate Ca2 + influx. Upon mild depletion of [Ca^2+^]_ER_ store, STIM2 can recruit and remodel STIM1 to form STIM1-STIM2 or STIM1-STIM1 complex, gating the ORAI1 promote Ca2 + flow into cytoplasm. Conversely, STIM2 can function as the inhibitor of STIM1-mediated SOCE via its interaction with CaM and rely on its variant STIM2.1 through the structure-allerosic combination compete with ORAI1. Cytosolic pre-STIM2 can also activate ORAI1 in a store-independent manner. Interaction of the cytosolic pre-STIM2 with TRPC1 facilitates its heteromerization with other TRPC proteins such as TRPC3 and VGC channel, directing further larger Ca^2+^ influx. In additon, STIM2 can serve as a scaffold protein bridging CAMKK2-AMPK interaction via its residues 349–441 and 442–746 as the combination sites, respectively, both of them located in the SOAR domain, which contributes to Ca^2+^-mediated AMPK activation. Additionally, the cytosolic SPF can combine with CaM via the conserved domain between Trp6 and Leu91 drive the promotion of CaM-dependent downstreams activation such as NF-κB transcription. Image made with BioRender.com

Cell homeostasis and activities highly rely on the Ca^2+^ concentration and signaling, as any deviations in baseline Ca^2+^ concentration can interfere with receptor-mediated Ca^2+^ signaling, as well as excessive rapid increases in cytosolic free Ca^2+^ have a clear association with the induction of cancer cell death [15, 16]. STIM proteins regulate store-operated calcium entry (SOCE) by sensing Ca^2+^ concentration in the ER (Fig. 2). However, the different properties and functions of STIM1 and STIM2 have been mostly described in vitro experiments and the STIM proteins family is ever-expanding [17–19]. Studies show that STIM2 knockout mice do not survive until adulthood, and RNA sequencing revealed 1424 differentially expressed genes indicating the critical function of STIM2 in cell fates [20].

### STIM2 regulate basal and homeostatic Ca2+ concentration

siRNA screening of the human signaling proteome identified STIM2 as a strong positive regulator of basal and homeostatic Ca^2+^ (Fig. 2). STIM2 knockdown in HeLa, HUVEC, and human embryonic kidney cells (HEK293) T cells, the decreased resting Ca^2+^ was detected in the ER and cytosol, while the hyperactive-like phenotype of STIM2a^-/-^ mutant zebrafish is caused by the dysregulation of Ca^2+^ homeostasis and signaling [21, 22].

#### ER Ca2+-store dependent Ca2+ influx

Due to the three amino acids difference in the cEF hand sequence which exhibits a 2-fold lower affinity to Ca^2+^ than STIM1 (400µM vs. 200µM), STIM2 can respond to minimal decreases in Ca^2+^. STIM2 also has a higher lipid-binding affinity than STIM1 does using PI (4,5)P2-containing liposome binding assays. This character favors the recruitment of STIM2-containing constitutively within ER-PM junctions mostly gating ORAI1 in immobile status which was driven by the inositol 1,4,5-triphosphate receptor (IP_3_R) function in resting conditions [21, 23]. The PM-anchoring STIM2 then traps STIM1 and triggers the remodeling of STIM1 C terminus, causing STIM1/ORAI1 couple in the open of ORAI1 channel that functions the tune fidelity of receptor-evoked Ca^2+^ signaling [24, 25]. In the model of NIH3T3, αT3 and HEK293 cells, STIM2 deletion reduced SOCE by more than 90%. Quantitative confocal fluorescence and fluorescence resonance energy transfer imaging demonstrated that in the absence of STIM2 expression, STIM1 did not translocate or/and with ORAI1 form punctae in PM-associated ER membrane (PAM) junctions following ER Ca^2+^ store depletion albeit STIM1 expression levels were unaffected [14, 26]. Contrastly, when STIM1 was knockout, the SIIM2-mediated Ca^2+^ signaling was sharply augmented via the PDGFR–PLCγ-IP_3_ signaling to maintain the myriad cell activities [27]. These results indicate that STIM2 is mandatory for STIM1 translocation and plays an essential role in their interaction with ORAI and the activation of SOCE [26].

However, when full depletion in ER Ca^2+^ store, STIM2 did not interfere with STIM1-mediated Ca^2+^ influx signals, which was observed in mast cells (RBL), T cells (Jurkat) and HEK293. Conversely, in this situation, STIM2 acts as the inhibitor of STIM1-mediated SOCE. In HEK293, PC12, A7r5, or Jurkat T cell lines, overexpression of STIM2 negates the STIM1-mediated SOCE opening [28]. Calmodulin (CaM) is activated upon the elevated cytosolic Ca^2+^, and then binds to the K-rich domain of STIM2 which shows low affinity (~ 1 µM). This occupation competitively blocks the interaction of STIM2 with the phospholipids of PM, thus prohibiting further Ca^2+^ influx. On the other side, one of the three STIM2 splicing variants, STIM2.1(STIM2.β) variant has an insertion of an extra amino acid sequence (383-VAASYLIQ-392) within the SOAR domain, which reduces the overall alpha-helicity and enhances the exposed hydrophobicity of the STIM2 CC domains albeit no global conformational change. So STIM2.1 is still Ca^2+^-dependent but can directly preclude ORAI1 channel activation by inducing structural perturbations in the STIM CC region. Studies show that splice-specific lipid-binding affinities within the SOAR region of STIM2.1 are lower compared to STIM2 (9.1 ± 2.7 nM vs. 40.7 ± 10.5 nM), but increased affinity towards CaM [29]. These results imply STIM2.1 negative feedback regulates ORAI1 activation on its unique structure-allergic combination with the PM lipid-binding and CaM [16, 30–32].

#### ER Ca2+ store-independent Ca2+ influx

The store-independent activation of Ca^2+^ influx is mediated by cell dialysis during whole-cell perfusion which is primarily regulated by the STIM2/CaM complex, of which CaM achieves its multi-talented functionality through two EF-hand domains, each is an independent capacity to bind Ca^2+^ and STIM2, respectively. The inhibition by CaM can be reversed by 2-aminoethoxydiphenyl borate (2-APB), resulting in rapid, store-independent activation of CRAC channels. The 2-APB-activated store-independent Ca^2+^ entry is mediated exclusively by endogenous STIM2. Using variants that either stabilize or disrupt intramolecular interactions of STIM C termini, the increased flexibility of the STIM2 C terminus was observed and the cytosolic pre-STIM2 can also activate ORAI1 in a store-independent manner [33]. This STIM2 C-terminus could be assembled to the nearby PM, recognize and contact with the PM-resided TRPC1, TRPC3, VGC channel or even with a chimeric ER membrane protein Kir6.2 [33–36]. In HEK 293 cells, STIM2 overexpression induces cytosol Ca^2+^ influx in a store-independent manner for the activation of nuclear factor of activated T-cells 1 (NFAT1) and nuclear factor-κB (NF-κB). NFAT1 is one of the members of the NFAT family. NFAT1 is dephosphorylated by Ca^2+^-dependent calcineurin and translocates into the nucleus for the transcription of downstream target genes, thus transmitting the Ca^2+^ signaling into gene expression. Both NFAT1 and NF-κB are involved in the transcriptional upregulation of T cell receptor (TCR)-stimulated target genes [36, 37]. Unlikely, the STIM1 variant failed to support its store-independent activation although it also has enhanced flexibility in the C terminus. The STIM1/STIM2 chimeric constructs indicated that coordination between N-terminal sensitivity and C-terminal flexibility is required for specific store-independent STIM2 activation. These results clarify the structural determinants underlying the activation of specific STIM isoforms, insights that are potentially useful for isoform-selective drug targeting [38].

#### Mitochondria-Ca2+ regulation

Mitochondria also act as Ca^2+^ stores for maintaining intracellular Ca^2+^ homeostasis. STIM2 is part of the mitochondria-associated ER membranes (MAMs) constructed by the ER-IP3R-voltage-dependent anion channel (VDAC)-mitochondrial Ca^2+^ uniporter (MCU). STIM2 serves as an inhibitor in mitochondrial Ca^2+^ uptake, which is mainly due to the predominant functional role of STIM2.1 described above. Contrastly, STIM2 knockout or inhibition can abrogate mitochondrial Ca^2+^ overload via reducing ER Ca^2+^ release, thus protecting against the trauma-induced ischemia/reperfusion injury in myocardia and neurons [39–42]. In skeletal muscle, STIM2 interacts with Calsequestrin 1 via its 92 N-terminal amino acids, and thus decreased SOCE and increased intracellular Ca^2+^ release, causing mitochondrial shape abnormalities [43].

### Other biological functions

Other biological functions aside from its function Ca^2+^ regulator, which is closely correlated with the structure of the cytosol domain or SPF of STIM2. STIM2 can interact with calcium/calmodulin-dependent protein kinase kinase 2 (CaMKK2) through its residues 349–441 of SOAR domain, while it combined with AMP-activated protein kinase (AMPK) via the region of residues 442–746 [44].

AMPK is a central energy-sensing kinase that helps cells survive under the low nutrient conditions, thus restoring metabolic homeostasis over time and space [45]. These results indicated that STIM2 acts as a scaffolding protein for the interaction of AMPK and CaMKK2. In this sense, STIM2 participates in the cell energy metabolism for the adaption to the surrounding environments. Additionally, the cytosolic SPF can combine with CaM via the conserved domain between Trp6 and Leu91 on the promotion of CaM-dependent downstream such as NF-κB transcription, while the Ca^2+^ concentration in the cytosol is unaffected [46]. CaM is a multi-functional cytosol molecule with a remarkable ability to interact with and regulate a plethora of structurally diverse targeted proteins [47]. These signal transductions by CaM via the STIM2 cytosol portion have revealed a remarkable repertoire of STIM2 actions and underscore the flexibility of STIM2 nature in cellular activities.

## STIM2 in the tumor and immune cells

### STIM2 abnormalities correlated with tumor cells malignant behaviors

STIM2 abnormality was involved in the pathogenesis of various types of tumors via its function on the cell cycle progression, metastasis and death resistance [48, 49] (Table 1). The low expression of STIM1 and STIM2 drives glioblastoma (GBM) progression [4, 50]. STIM2 overexpression was observed in colorectal cancer (CRC) which was correlated with poor prognosis [36, 51]. Silence of ORAI1/STIM2 in the cells shows weaker invasive and migratory potential but exhibits accelerated proliferation and apoptosis resistance. In ER-positive breast cancer cells, STIM2, as well as TRPC6 and ORAI3 were involved in cytosolic Ca^2+^ signaling [52]. STIM1 and STIM2 were upregulated in MCF-7 breast cancer cells, which enhance the migration and invasiveness ability via STIM2-NFAT1-TGFβ1 pathway promotion on its epithelial-mesenchymal transition (EMT) phenotype [53]. Similarly, STIM2 overexpression favors melanoma cell migration and invasive, which can be prohibited due to the C313 being sulfonylated by reactive oxygen species (ROS) that block STIM2 oligomerization and gating ORAI [54]. However, STIM2 can be involved in an ORAI-independent manner. In cholangiocarcinoma, STIM2 suppresses tumor metastasis in vivo by reducing keratin 8 [55]. In CRC, STIM2 loss leads to the increase of SERCA2-dependent ER Ca^2+^, and then activates c-Myc and the PERK/ATF4 branch of ER stress facilitating the increased protein translation and transcriptional and metabolic rewiring which support increased tumor size, invasion, and metastasis [56].

**Table 1:** STIM2 in tumor and immune cells

Cell type	Sample	STIM protein expression	Biological function	Molecular mechanism	Ref.
**Cancer cell**					
Breast cancer	cell line	STIM1 ↑ STIM2 ↑	promote EMT, migration and invasion	NFAT1-TGFβ1 pathway, TGF-β induced EMT, increased SOCE, STIM2 induced non-SOCE	[54]
Cholangiocarnoma	cell line, human tumor tissue	STIM2 ↑	inhibit cell metastasis	KRT8(keratin 8) reduction, non-SOCE	[56]
CRC	cell line	STIM1 ↑ STIM2 ↓	promote tumorigenesis, metastasis and apoptosis resistance	reduced Ca^2+^ stores, increased resting Ca^2+^, SOCE and ISOC, increased ORAI1, ORAI2, ORAI3 and TRPC1	[51]
CRC	---	STIM1 ↑ STIM2 ↓	regulate cell death and drug resistance	disturbances in Ca^2+^ homeostasis, STIM-mediated ORAI1-independent apoptosis, STIM1/ORAI1/TRPC1 driven SOCE	[52]
CRC	cell line	STIM2 ↓	promote tumorigenesis, invasion and metastasis	increased SERCA2-dependent ER Ca2+, activated c-Myc and PERK/ATF4 branching, increased protein translation, transcription and metabolic reorganization,	[57]
ER-positive breast cancer	cell line	STIM2 ↑	promote proliferation tumorigenesis and metastasis	STIM2-ORAI3, STIM2-TRPC6, cytosolic and ER Ca^2+^ homeostasis in resting conditions, ER stress	[53]
GBM	---	STIM2 ↑	promote tumorigenesis and metastasis	imbalance of Ca^2+^ homeostasis, disruption of BBB integrity	[4]
Melanoma	cell line	STIM2 ↑	promote cell migration and invasion	ROS, C313 STIM2-ORAI	[55]
Primary GBM	cell line, human tumor tissue	STIM2 ↑	promote tumorigenesis and metastasis	TNFSF13B, COL4A2, gene dosage, PI3K/AKT pathway	[50]
**Immune cell**					
CD4^+^ helper T cell	C57BL/6 mouse model	STIM1 KO	impaired SOCE, reduced cytokine production	STIM/ORAI-mediated Ca^2+^ influx, NFAT-mediated transcription of downstream genes (IL-2, IL-4, IFN-γ), CRAC current impairment STIM/ORAI-regulated development and function of T cell	[67]
		STIM1 & STIM2 KO	Severely/completely impaired SOCE, reduced cytokine production, notable lymphoproliferative phenotype		
CD4^+^ helper T cell	C57BL/6 mouse model	STIM1 KO	severely impaired SOCE, reduced cytokine production	STIM/ORAI-mediated Ca^2+^ influx, NFAT-mediated transcription of downstream genes (IL-2, IL-4, IFN-γ), CRAC current impairment STIM/ORAI-regulated development and function of T cell	[68]
		STIM2 KO	impaired IL-4 production		
		STIM1 & STIM2 KO	perinatal death		
leukemic T lymphoblast	mouse model	STIM1 and STIM2 KO	completely impaired SOCE, significantly prolonged survival	STIM/ORAI-mediated Ca^2+^ influx, SOCE -dependent inflammation, downregulated inflammation-related signaling pathways and proinflammatory Genes Expression (TNF-a, IL-10, IL-16, IL-22, CCL6, IFN-α, IFN-β)	[103]
B cell	mouse model	STIM1 and STIM2 KO	completely impaired SOCE, cell proliferation and regulatory function	Antigen /BCR complex, STIM/ORAI-mediated Ca^2+^ influx, NFAT-mediated transcription of downstream genes gene IL-10	[69]
macrophage	C57Bl/6 mouse model	STIM1 KO	severely impaired SOCE, defective phagocytosis, normal cytokine production and chemotaxis	GPCR/TLR4 complex, STIM1/ORAI-mediated Ca^2+^ influx, NFAT-mediated transcription of downstream gene (TNF-α, IL-6, IL-1β), C5a/IgG-induced FcγR-mediated phagocytosis, FcγR-mediated Ca^2+^ response	[70]
		STIM2 KO	modestly impaired SOCE, defective phagocytosis, reduced cytokine production and chemotaxis		
neutrophil	mouse model, human dHL60 cell line	STIM1 KO STIM2 KO	impaired SOCE, reduced chemotaxis impaired SOCE and bactericidal function	NADPH oxidase, phagosome maturation, cell polarization, receptor families (TLRs, FcR, integrin), STIM/ORAI-mediated Ca^2+^ influx, superoxide release by PKCα/β, ROS production, degranulation, phagocytosis	[71]
	mouse model				
Neutrophil basophil	mouse model	STIM2 KO	modestly impaired SOCE, minimally impaired bactericidal response, significantly reduced cytokine production	STIM2/ORAI-mediated CA^2+^ influx, SOCE regulated NF-κB activation, IKKα/β, CAMKII, ROS production, degranulation, phagocytosis, ROS-mediated inhibition of cytokine production, cytokine-mediated inflammation (TNFα, IL-10, IL-6, IFN-γ) stimulus dependent IL-4 production (IgE-driven STIM1 response, IL-3/ IL-33-driven STIM2 response), IgE-mediated chronic inflammation, STIM/ORAI-mediated Ca^2+^ influx, distinct time courses of Ca^2+^ influx	[72]
		STIM1 KO	reduced inflammation		

↓ indicates that the protein is down-regulated in the sample of study model

↑ indicates that the protein is up-regulated in the sample of study model

### STIM2 regulate the short-term and long-term function of immune cells

**Fig. 3:**
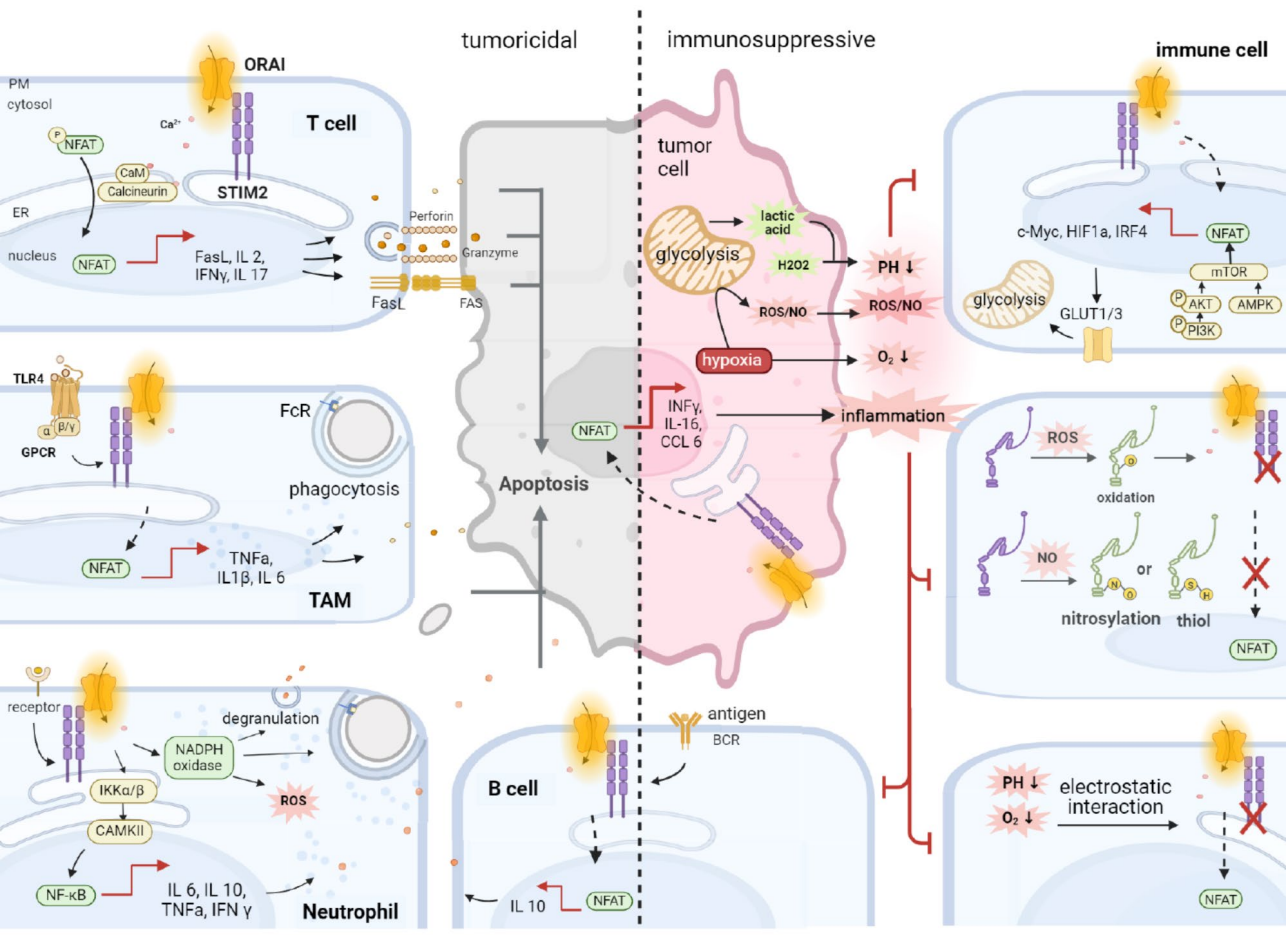
STIM2 in the tumor and immune cells of TME. **Left**: STIM2 mediated the tumor cells-killing function of immune cells. In T cells, STIM2-mediated SOCE promotes FasL expression, lytic granules release and cytokines production, inducing tumor cell apoptosis. In both TAM and neutrophil, STIM2-mediated SOCE promotes phagocytosis, various kinds of cytokines production and tumor cell apoptosis. Additionally, STIM2 can foster neutrophil degranulation and ROS production via NADPH oxidase. In B cells, STIM2-mediated SOCE promotes IL-10 production and tumor cell apoptosis. **Central**: Cytokines of INFγ, IL6 and CCL6 from tumor cells reshape IME and its ingradients. **Right**: TME modify STIM2 of immune cells. Upper: STIM2 mediate NFAT activation, in conjunction with PI3K-AKT-mTOR and AMPK regulating GluT1/3 faciliating glycolysis for nutrition supply which is required for T cells function. *Middle*: The ROS/NO in TME permeably influx into cytoplasm modify STIM2 residues causing the impairment of immune cells. *lower*: Acidic TME and hypoxia inhibit the STIM2-ORAI due to high sensitivity of STIM2 to the electrostatic interactions. Image made with BioRender.com

Immune cells include adaptive immune cells (T and B cells), and innate cells (macrophages, neutrophils, dendritic cells (DCs), innate lymphoid cells, myeloid-derived suppressor cells and NK cells) [57]. Intracellular Ca^2+^ signaling is a fundamentally important regulator of immune surveillance, immune self-stability and immune defense in these cells [21] (Fig. 3). STIM1 and STIM2 synergize the optimal Ca^2+^ oscillations, but STIM2 expression is necessary to maintain the frequency of Ca^2+^ oscillations, as well as in the coupling of ORAI1-mediated Ca^2+^ influx to NFAT1 activation (Fig. 3). STIM2 knockdown in HEK293 cells diminishes agonist-induced Ca^2+^ signaling and nuclear translocation of NFAT1. STIM2 recruits STIM1 to ER-PM junctions, which induce the congregation of the A-kinase anchoring protein 79 (AKAP79), a scaffolding protein, to form a signaling complex that gating ORAI1 channel, and enables Ca^2+^ nanodomains near open ORAI1 channels to selectively drive NFAT1 activation with high fidelity [58]. STIM2 knockdown disrupts the formation of the ORAI1/STIM1/AKAP79 complex, while the missense mutation of STIM1 only attenuated this association whereas that can be recovered by co-expressing STIM2 in these cells [59, 60]. These results clarify the fundamental contribution of STIM2 proteins to the spatiotemporal pattern of Ca^2+^ signaling of immune cells for the regulation of the target gene expression which determines the cellular process related to cell proliferation, differentiation, maturation and survival, and the short-term function of immunological synapse (IS) formation and cytotoxic granules release [10] (Table 1).

#### T lymphocytes

T lymphocytes are one of the most immune cells in the human body. It includes heterogeneous subsets but shows cooperative function on the immune attack. Naive CD4^+^ T cells have high STIM2.1 expression, knockdown of which leads to the remarkable increase of SOCE activity and Ca^2+^ ion level [61]. In the CD8^+^ T cells, when its CD28 binds to CD80/CD86 of antigen-presentation cells (APC), it exhibits significant cytoplasm Ca^2+^ influx that is largely dependent on the STIM2 activity independent of ER Ca^2+^ store, and this process could be abolished via the competitive block by Cytotoxic T Lymphocyte-Associated Antigen-4 (CTLA-4) [62]. Mechanistically, STIM2 gates CRAC channel opening Ca^2+^ influx for calcineurin-dependent NFAT1 nuclear translation to conjunction with PI3K-AKT-mTOR, a kind of nutrient-sensing pathway, which was induced for the facilitation of the gene expression regulating glycolysis and oxidative phosphorylation to provide adequate nutrient supply for T cell [63]. This process is critical for supporting the clone expansion and migration of T cells, including CD4^+^ T helper cells, CD8^+^ cytotoxic and natural killer(NK) cells, and their adaptive immune response to anti-tumor [64]. CD8^+^ cytotoxic T cells are the prime killers, which fight against tumor cells mainly via cytolytic granules release, cytokines secretion and FasL expression that are highly dependent on the expression and activity of STIM2 [65]. STIM1 and STIM2 knockdown in T lymphocytes of mice exhibit compelling reduction of cytokine production and antigen-dependent proliferation. However, in the cells with only homozygous STIM1 deletions, the cytotoxic activity of CD8^+^ T cells is largely preserved, while if the STIM1 and STIM2 were both knockdown, severe deficiency in its polarization and cytokine production and lytic granule exocytosis was observed [66, 67]. STIM2 mediated the NFAT1 nuclear translocation for promoting the expression of its downstream targets including FasL, IFN-γ, interleukin (IL)-2, IL-17 expression that interprets and accentuates the key role of STIM2 in the T cells on tumor cells-killing [65].

#### B lymphocytes and other immune cells

STIM2-mediated Ca^2+^-related activation of NFAT1 and calmodulin–calcineurin kinase is also required for the development, survival and function of other immune cells in responses to numerous stimuli. In vitro experiments, knockdown with STIM1 and STIM2 can completely inhibit B cell receptor -mediated B cell proliferation, survival and IL-10 production via the decrease of BCL-2, mTORC1 and c-Myc [68]. In macrophages, STIM2, independent of STIM1 mediates the IgG-mediated phagocytosis induced by C5a on FcγR, the cell migration stimulated by G protein–coupled receptor, and the TNF-α production by Toll-like receptor 4 [69]. In neutrophils, STIM1 and STIM2 are in unity to regulate neutrophil Ca^2+^ signaling. Further study indicated that STIM1 functions dominantly in the bactericidal functions for the promotion of ROS production, degranulation, and phagocytosis [70], while STIM2 is at least in part responsible for redox regulation of cytokine gene expression [71]. In vivo loss of STIM2 results in decreased cytokine levels and protection from mortality in a mouse model of systemic inflammatory response syndrome [72]. In basophils, rapid and STIM1-dependent Ca^2+^ influx occurs when strong FcR stimulation, while if cytokine receptors (IL-3R and IL-33R) activation, STIM2 was exclusively activated and responsible for the secretion of IL-4 cytokine. DCs predominantly express STIM2 which can be recruited to the IS upon the intercellular adhesion molecule 1 (ICAM-1) stimulation, but only very low levels of STIM1 [73].

Taken together, STIM2 has a distinct role that is major in the promotion of cytokines and/or other cytotoxic molecules expression and release, and this function is severely impaired when it is knockdown with siRNA or pharmacological inhibitor even in the condition of intact STIM1.

## STIM2 function on the immunomodulation of TME

The TME mainly consists of tumor cells and immune cells. It also includes the soluble factors including ROS, reactive nitrogen species (RNS), cytokines and even characteristics of nutrient deficiency, hypoxia and activation, which contribute to the reshaping of the immune microenvironment and mostly confer immune resistance. STIM2 could be the transmitter in the well-buffered cytosol that allows the physico-chemical molecules penetrated into the immune cells [74]. Low levels of Ca^2+^-related genes expression in high-risk score cases were accompanied by worse outcomes in glioma patients, hinting that Ca^2+^ signaling pathway may be a therapeutic target to reverse immunosuppressive micro-environment and improve the effect of immunotherapy in glioma [75]. STIM2 abnormality was closely correlated with immune TME indicating of poor prognosis, and the dysregulation of STIM2 in the stroma region was associated with the more invasive clinical phenotype and poor outcome in prostate cancer [76, 77] (Fig. 3).

### STIM2 facilitates glycolysis of immune cells

When the antigens bind to the TCR teamed with the simultaneous co-stimulatory signals such as CD28, naïve T cells undergo extensive proliferation, growth and differentiation into effector T cells (Teff) which are highly dependent on glycolysis [78]. A recent study shows that SOCE/STIM2 directs the metabolic reprogramming of naive T cells by regulating the expression of glucose transporters (GLUT1 and GLUT3), glycolytic enzymes, and metabolic regulators through the activation of nuclear factor of activated T cells c1 (NFATc1, also named NFAT2) and the PI3K-AKT kinase-mTOR nutrient-sensing pathway on the promotion of the c-Myc, IRF4, HIF1a, and other transcription factors into the glycolysis statue of effector. These processes of STIM2-mediated glycolysis are also observed in CD4^+^ T cells [63, 79]. On the other side, the glucose argument is the expression levels of ORAI1, ORAI2, and STIM2 that form positive feedback for cell glucose absorption and glycolysis [80]. Abolishing SOCE in mouse T cells by conditional deletion of STIM2 impaired TCR-induced proliferation in vitro, as well as its clonal expansion and cytotoxicity function in vivo [63]. Therefore, it is proposed that STIM2 expression is a key checkpoint at which T cells access adequate nutrient supply to support their clonal expansion and adaptive immune responses. But there usually exists limited glucose due to it being largely consumed by the tumor cells on the requirement for its high proliferation. In this sense, SOCE/STIM2 prime the glucose metabolism of T cells that competes with its neighbor tumor cells, and the abnormalities of STIM2 or its downstream will render immune suppression resulting from T dysfunction. Multiple types of normal cells can regulate STIM1 and STIM2 as a defense mechanism against low energy availability [81]. STIM2 deficiency in T cells will cause serious function impairment due to the glycolysis processes requiring continuous high activity of NFAT1 and NFAT2 by TCR-mediated store-operated Ca^2+^ entry to induce efficient expression of downstream genes of NFAT and NFAT-target transcription factors [79].

### STIM2/AMPK regulates metabolic fitness of immune cells

AMPK is a key molecule in cell energy homeostasis, it can be activated in CaM-mediated and STIM2-dependent way. AMPK senses the change of intracellular energy and is activated to initiate the response of different types of cells to diversified energy stress to restore energy homeostasis [82]. Studies show that AMPK helps effector T cells (Teff) in their metabolic adaptation in response to reduced glucose availability [83]. Increasing AMPK activity also orchestrates oxidative metabolism, proliferation, and in vitro recovery of human CD4^+^ T cells. AMPK increases glycolytic rates via the activation of mTOR in favor of the M1 anti-tumor phenotype in macrophages. AMPK abnormalities cause the deceased fitness and exhausted status of multiple immune cells [82]. The metabolic fitness of immune cells is pivotal for anti-tumor immunity, thus effectuating the STIM2 activity on AMPK signaling in favor of optimal immuno-environment in the anti-tumor armamentarium.

### ROS/nitric oxide (NO) modifies STIM2 in immune cells

ROS or/and NO usually enrich in TME due to the nourishing metabolism of both immune cells and tumor cells. The reactive cysteine residues that are the major targets of ROS and NO, and modified cysteines are emerging post-translational modifications (PTMs). The PTMs refer to a protein by means of modified chemical groups covalently attached to some of its amino acid residues [84]. PTMs participate in the extensively physiological and pathologic processes, capable of altering the function, conformation, or localization of proteins [85].

STIM2 protein has 10 additional cysteine residues in the C terminus facing the cytoplasm in comparison to STIM1, and most of them are critical for the allosteric effector of STIM2 for Ca^2+^ signaling. These residues are liable to be modified in the redox environments [86]. It was reported that C313 in STIM2 can be oxidated and thereby inhibit its oligomerization in melanoma cells [54]. Interestingly, in the ROS-induced aging study, the oxidation of C313 residue of STIM2 has been detected in 9 of 20 aged mice [87]. These results indicate that STIM2 oxidative modifications are pathophysiology highly relevant. Therefore, it is tempting to ascertain that the ROS from TME into the well-buffered cytosol near the PM in immune cells can regulate the intermolecular thiol bridge formation involving the cysteines presented in the cytosolic tail of STIM2, but whether these modulations inhibit or promote the extension/activation of STIM2 molecule for its gating ORAI channels are not yet fully understood. Noticeably, this oxidation can also lead to intra- and inter-molecular cross-link and trap of proteins in multimolecular complexes such as STIM2-STIM1 that could affect their oligomerized activation and thus the fine-tuning of intercellular Ca^2+^ signaling was mostly undermined [88, 89].

Similarly, due to the presence of additional cytosolic cysteines, STIM2 is also highly sensitive to S-nitrosylation. It is reported that C112, C150 and C157 cysteines of STIM2 are thiol modification or nitrosylated under nitrosative stress [90]. These modifications stabilize the EF-SAM domain thermodynamically and then prevent STIM2 activation and the subsequent SOCE function. The NO donor nitrosoglutathione (GSNO) thermodynamically stabilizes the STIM2 Ca^2+^ sensing region in a cysteine-specific manner [90]. In HEK293T cells, enhanced free basal cytosolic Ca^2+^ and SOCE mediated by STIM2 overexpression could be attenuated by GSNO or the mutation of the modifiable cysteine located in the luminal domain [90].

Additionally, ROS causes increased levels of oxidized glutathione which can interact and make C56 S-glutathionylation located in the EF-SAM hand of STIM1, provoking STIM1 constitutive oligomerization gating for ORAI activation [91]. This reactive cysteine is conserved in STIM2 that complex the performance of ROS on the Ca^2+^ influx and signaling. Therefore, the effects of ROS/NO on the STIM2 of immune cells depend on a wide array of covalent modifications, as well as the duration and the degree. It is reported that moderate ROS levels allow for the activation, signaling and differentiation of T cells, whereas high levels of ROS lead to T-cell exhaustion. This discrepancy was observed in the EPI-induced cell changes, such as T cells presented increased SOCE and increased ROS production at 30 min after EPI treatment, but SOCE was indeed significantly reduced in T cells treated with EPI for 6 h or longer [92]. Ca^2+^-dependent activation of NF-ΚB, involving IKKα/β mediator or/and the phosphorylation of calcium/calmodulin-dependent protein kinase II (CAMKII) is the underlying mechanism of ROS on the immune cells function [93].

### Hypoxia and acidic environment inhibit STIM2 activation in immune cells

Hypoxia is a relatively common event in TME because tumors usually grow beyond the capacity of the vasculature in the provision of sufficient oxygen to the tumor cells. The highly glycolytic tumors release abundant lactate, so extracellular acidosis is a hallmark of tumor progression. TME-resided immune cells also exhibit intracellular acidification due to hypoxia [94, 95]. STIM2 is sensitive to a range of hypoxia, to which it can mediate appropriate pathophysiological responses. For example, STIM2 deficiency decreases zebrafish survival under hypoxia conditions [20]. The overexpression of STIM2 was observed in rat pulmonary arterial smooth under chronic hypoxic conditions. STIM2-knockdown neurons are protected from hypoxic cell death [96, 97]. Similar to other CRAC components such as ORAI1/2/3 and STIM1, acidic pH could inhibit the multiple functions of STIM2. Experiments show that the titratable residues such as cysteine, histidine and glutamic acid can be involved in internal pH sensing in TRPV1 or HCN1, and possibly in the STIM2. The pH-mediated inhibition of SOAR-ORAI interaction reflects electrostatic interference by H^+^ ions. Furthermore, pH-mediated inhibition of STIM2-ORAI1 was observed in fluorescence resonance energy transfer imaging, indicating a greater dependence for STIM2 than STIM1 on electrostatic interaction [98]. Hypoxia and acidification induce the anergy of CD8^+^ T cells, mostly due to the decrease of cytokines, including IL-2, TNF-α and IFN-γ [37, 99–101]. These observations and the prior role of STIM2-mediated cytokines release suggest that STIM2 is the equitable target of hypoxia and acidity attributed to the turbulence of cellular Ca^2+^ signaling.

### Tumor STIM2-mediated cytokines reshape immuno-environment

Cytokines are one of the most important intermediates for the counter-acted impact of tumor cells on the function of immune cells within TME. In leukemic T lymphoblasts, cytokines and chemokines including INFγ, IL-16 and C-C motif chemokine ligand 6 (CCL6) were released, which are the chemoattractants inducing the movement of tumor-associated macrophages (TAMs) toward the tumor-infiltrated tissues, and these molecules largely rely on the expression and function of STIM1 and STIM2 in the infiltrated tumor cells. When STIM2 was depleted in mice, the production of TNF-α, IL-1β and IL-6 was blocked, thus dampening the tumor-infiltration associated inflammation [102]. Similarly, in (micro)glial cells, STIM2 activation promotes the release of inflammatory cytokines which seriously damage neurons [103]. It is postulated but still needs further investigation if and how tumor cells with STIM2 abnormalities impacts the immune cellular activities and functions.

## Conclusions and perspectives

**Fig. 4:**
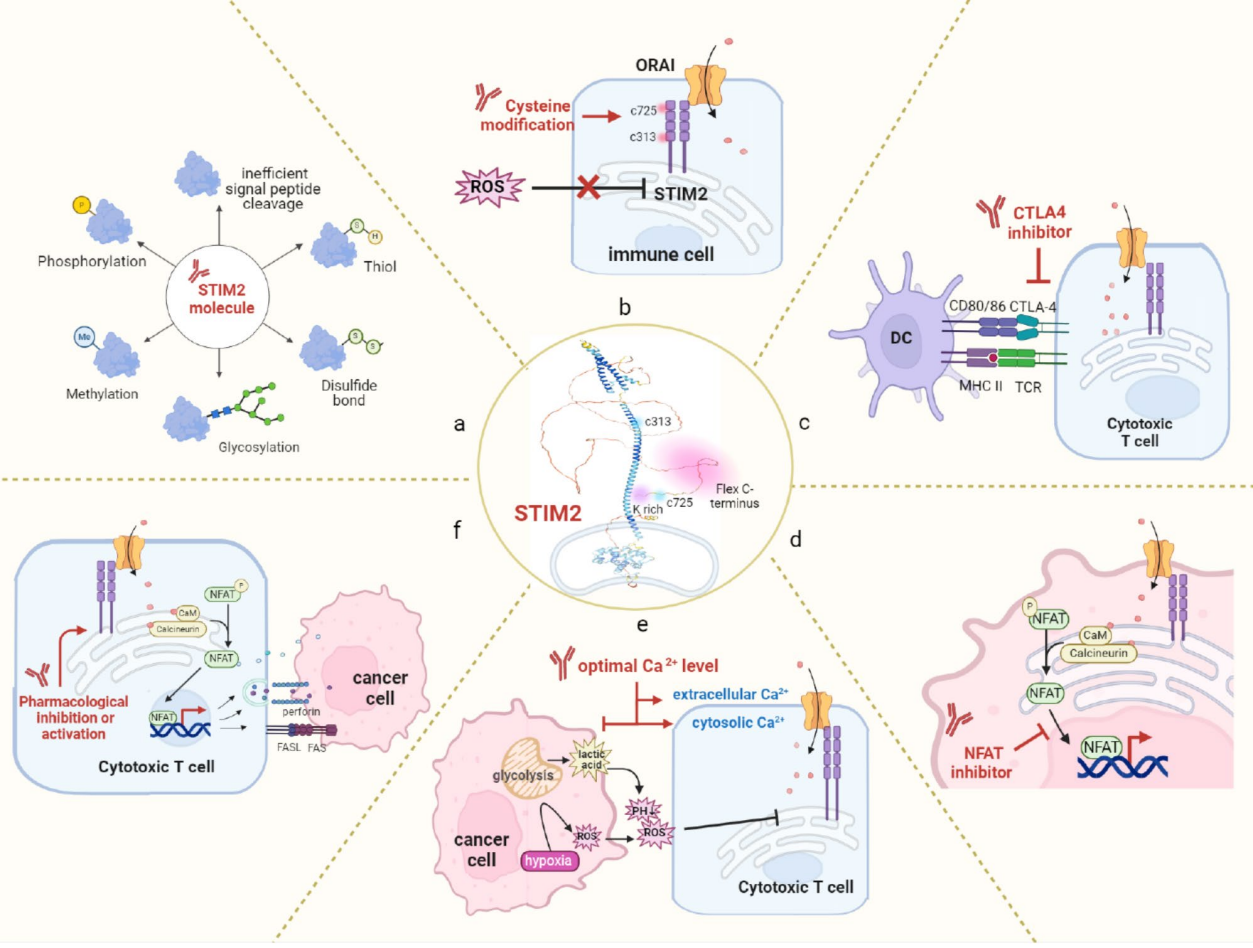
The opportunity and perspective of STIM2 as a target in anti-tumor therapeutic strategy. **a**: Full characterization of cytosolic structure for epigenetical modification. **b**: Pharmacological intervention on the flexible C-terminus domain (CTD) which as an adaptor or heterodimer with various molecules. **c**: Modulating the ratio of splicing or/and pre-protein variants for the optimal Ca^2+^ level. **d**: NFAT as therapeutic substitute with tumor-targeted delivery system. **e**: Cysteine alteration abolish ROS effect on immune suppressive TME. **f**: Co-target with CTLA-4 in synergistic immunotherapy combination

Due to the big leap and rapid evolvement of immunotherapy and its efficacy as a single agent in multiple types of tumors, the advent endeavor is focused on how to magnify its effects or overcome its resistance (Fig. 4). Except for the intrinsic character of tumor cells, other resources of immunotherapy resistance commence from the complex interaction between tumor cells and IME. Tumor cells educate immune cells to the blunt phenotype mainly via their metabolites or signaling crosstalk. Therefore, identifying molecules that act as focal points linking this connection could be a promising strategy to fight tumors. In this scenario, STIM2 acts as the nexus between cellular Ca^2+^ homeostasis, energy fitness, CaM signaling network and anti-tumor immunity (Fig. 4).

Epigenetic modification of the STIM2 with the substituting reagents/chemical compounds abolishes its susceptibility to the TME components, or the alterations of these components could offer substantial therapeutic meanings [104]. These approaches would also aid in the formulation of novel remedial regimens that be synergistically integrated into the CTLA-4-targeted immunotherapy or that STIM2 expression could be collated to the current biomarkers for enriching the beneficial population. The function of CD8^+^ T cells cytotoxicity is largely dependent on STIM2-mediated basal and sustainable Ca^2+^ signaling that drives NAFT nuclear translocation for the expression of target genes. Pretty low cytosolic Ca^2+^ (122–334 nM) and external Ca^2+^ (23–625 µM) were observed in the requirement for the maximum function of T cells including CD8^+^ cytotoxic T cells, CD4^+^ T cells and NK-T cells [105, 106]. It is reported that low serum Ca^2+^ may give SLE patients an enhanced cellular immune status [107]. Therefore, a delineated mechanistic exploration of STIM2-mediated regulation in immune cells and thus subsequent IME in assorted types of tumors will largely broaden our view and knowledge.

In turn, STIM2-mediated Ca^2+^ signaling or the multifaceted Ca^2+^-related molecule CaM promotes the release of a bunch of inflammation cytokines or other signals intersected with the IME, however, there are numerous evidences demonstrate the dual nature of STIM2 in tumor cells and the resultant surroundings [108, 109]. Further research on the characterization of the Ca^2+^ toolkits on the tumor type, tissue site and treatment intervention should be warranted, for the elucidation on the role of Ca^2+^ ion that is the pro- or anti-tumorigenic functions of STIM2 [56]. Although the experiment results are still in its burgeoning stage, Ca^2+^ remodeling or STIM2-targeted small molecule inhibitors offer the opportunity for this conceptualization [110, 111]. These researches will pertain to the context-special role of STIM2 using human tissue samples, patient-derived xenograft or three-dimension models of various types of tumors [99, 112]. The other way in more in-depth mechanistic research is to better define the downstream effectors of STIM2. As above-mentioned, NFAT1 and NFAT2 activation denote the STIM2 role and thus NFAT-targeted approach could be an alternative therapeutic approach for STIM2. NFAT nuclear translocation determines their active function as transcription factor on the targeted genes, and the underlying mechanism on the regulation of this process could provide novel perspective strategies for drugs-development [113, 114].

Collectively, STIM2 has been proposed as a relevant player in pathological conditions related to cancer, aging and nervous diseases. The bolstering roles on the Ca^2+^ homeostasis, subsecond Ca^2+^ microdomains, pertinent NFAT1 or/and NFAT2 transcription factor function and non-Ca^2+^ dependent CaM kinase activation has clarified the complex but still interesting role of STIM2 in reshaping immune cells metabolism on themselves or the from the crosstalk with tumor cells [115]. Today, our ability to pharmacologically dissect and manipulate the STIM2 signaling pathway remains a readily accessible way to expand our knowledge in this field, while molecular biological strategies are difficult to employ. However, it is still promising that novel technology such as three-dimension spatiotemporal simulations or computational modeling could break new ground for the discovery of alternative and attractive SITM2-targeted therapeutic approach [116–118].

Generally, unlocking the potential of the TME can optimize the immunotherapy efficacy [119, 120]. STIM2 and its variants are the generalists on immunomodulation, due to the updated novel recognition of its structure and function as the components of tumor-TME niches. Therefore, STIM2 is becoming the promising therapeutic target for dual targeting the tumor-TME which should improve cancer control and clinical outcomes. Certainly, the time spatial heterogeneity, tissue-type and context-dependence should be considered in the future for the STIM2 study [121].

## Data Availability

Not applicable.
